# 5-[(4-Methyl­phen­yl)sulfon­yl]-1-phenyl­thio­pyrano[4,3-*b*]indole-3(5*H*)-thione di­chloro­methane monosolvate

**DOI:** 10.1107/S2414314623003541

**Published:** 2023-05-12

**Authors:** Benjamin Dassonneville, Dieter Schollmeyer, Heiner Detert

**Affiliations:** a University of Mainz, Department of Chemistry, Duesbergweg 10-14, 55099 Mainz, Germany; Goethe-Universität Frankfurt, Germany

**Keywords:** crystal structure, sulfur, annulated heterocycles

## Abstract

Rhodium-catalyzed [2+2+2] cyclo­addition of carbon di­sulfide to *o*,*N*-dialkynyl­tosyl­anilines gives two isomeric indolo­thio­pyran­thio­nes, a violet and a red isomer. This is the first crystal structure of a red isomer, which crystallizes with one solvent mol­ecule of di­chloro­methane in the asymmetric unit, C_24_H_17_NO_2_S_3_·CH_2_Cl_2_. In the extended structure, centrosymmetric pairs of the planar annulated system are arranged in strands and solvent mol­ecules fill the space between the strands.

## Abstract 2

Rhodium-catalyzed [2+2+2] cyclo­addition of carbon di­sulfide to *o*,*N*-dialkynyl­tosyl­anilines gives two isomeric indolo­thio­pyran­thio­nes as violet and red isomers. This is the first crystal structure of a red isomer, which crystallizes with one mol­ecule of di­chloro­methane in the asymmetric unit.

## Graphical abstract

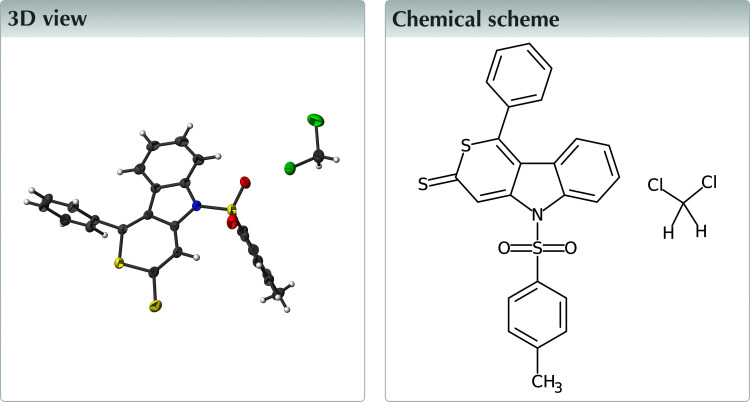

## Structure description

**Figure 1:**
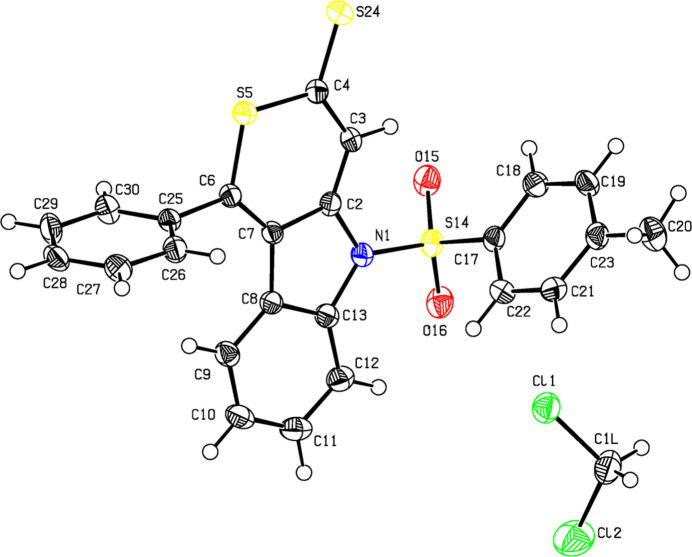
View of the title compound. Displacement ellipsoids are drawn at the 50% probability level.

**Figure 2:**
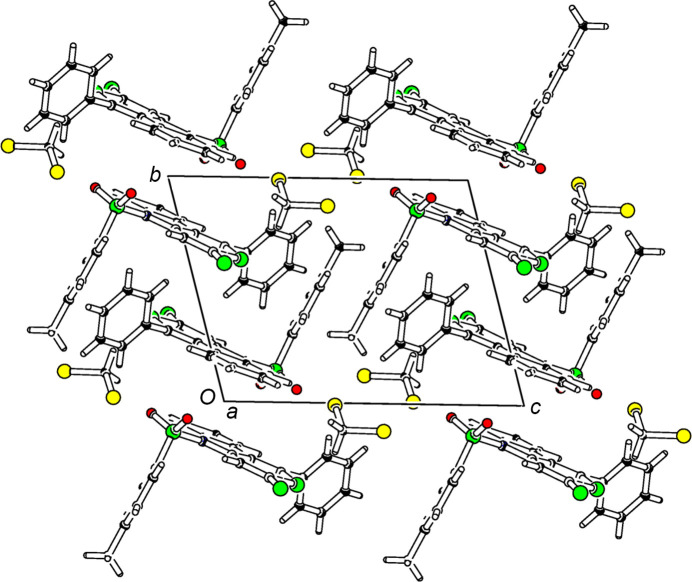
Part of the packing diagram, viewed along the *a* axis.

Transition-metal-catalyzed [2+2+2] cyclo­additions are an atom-economic route to aromatic rings (Reppe *et al.*, 1948 ▸; Bönnemann, 1978 ▸; Vollhardt, 1984 ▸). With tethered diynes, annulated systems are accessible, *e.g.* carbazoles and carbolines (Heller & Hapke; 2007 ▸; Dassonneville *et al.*, 2011 ▸). The first thio­pyran­thione was reported in 1973 (Wakatsuki & Yamazaki, 1973 ▸), followed by rare examples of this heterocycle. The [RhCl(C_8_H_14_)_2_]_2_–BINAP [BINAP is 2,2′-bis­(di­phenyl­phosphan­yl)-1,1′-binaphth­yl] (Tanaka *et al.*, 2006 ▸) catalyzed [2+2+2] cyclo­addition of carbon di­sulfide to *o*,*N*-di­alkynyl­tosyl­amides gives mainly the violet indolo­thio­pyran­thio­nes with a [3,4-*b*] annulation, in some cases accompanied by their red isomers differing in the annulation pattern (Dassonneville *et al.*, 2023 ▸). While the structure of the violet indolo­thio­pyran­thio­nes has been proven exemplarily in a single-crystal XRD study (Dassonneville *et al.*, 2010 ▸), the structures of the red isomers were hitherto only based on spectroscopic data. This report gives the first crystal structure of a red isomer. The moderate stability of the red thio­pyran­thione allowed crystals to be grown by slow evaporation of a solution in di­chloro­methane/petroleum ether. The title compound (Fig. 1 ▸) crystallizes with one mol­ecule of the solvent. Centrosymmetric pairs with a distance of 3.5556 (13) Å between the centroids of the N1/C2/C7/C8/C13 and C8–C13 π-systems are arranged in strands along the *a* axis (Fig. 2 ▸). The solvent mol­ecules fill the volume between the strands. The heterocyclic framework is essentially planar, the maximum deviation from the mean plane of the π-system is 0.043 (2) Å at the thio­carbonyl C4 atom. With a dihedral angle of 82.44 (8)°, the phenyl ring is close to being orthogonal to the fused-ring system. The tolyl ring is also almost perpendicular [dihedral angle = 83.08 (8)°] to the plane of the three-membered ring system. The N—S—C angle of the sulfonyl group is 103.79 (9)°. The C—N bonds in the pyrrole ring are significantly different, with the N–phenyl bond [1.436 (3) Å] significantly longer than the N–thio­pyrane bond [1.405 (3) Å]. This and alternating bond lengths between the indole-N atom and the thio­carbonyl are an indication of an electronic coupling between the nitro­gen thio­carbonyl group. Other structural features of the tricyclic core are similar to those of the isomeric system with a methyl instead of phenyl substitutuent. The two isomers differ in color and in the relative position of the indole-N atom to the thiocarbonyl group. In the violet isomer, these units are in perfect conjugation whereas the *meta*-conjunction in the red isomer restricts electronic interaction, thus shifting the absorption maximum about 60 nm to higher energies.

## Synthesis and crystallization

The synthetic and spectroscopic details for the title compound have been reported previously (Dassonneville *et al.*, 2023 ▸).

## Refinement

Crystal data, data collection and structure refinement details are summarized in Table 1 ▸. H atoms were placed at calculated positions and refined in the riding-model approximation, with aromatic C—H = 0.95 Å, methyl­ene C—H = 0.99 Å and methyl C—H = 0.98 Å, and with *U*_iso_(H) = 1.5*U*_eq_(C) for methyl H atoms and 1.2*U*_eq_(C) otherwise.

## Supplementary Material

Crystal structure: contains datablock(s) I, global. DOI: 10.1107/S2414314623003541/bt4137sup1.cif

Structure factors: contains datablock(s) I. DOI: 10.1107/S2414314623003541/bt4137Isup2.hkl

Supporting information file. DOI: 10.1107/S2414314623003541/bt4137Isup3.cml

CCDC reference: 2077250

Additional supporting information:  crystallographic information; 3D view; checkCIF report

## Figures and Tables

**Table 1:** Experimental details

Crystal data	
Chemical formula	C_24_H_17_NO_2_S_3_·CH_2_Cl_2_
*M*_r_	532.49
Crystal system, space group	Triclinic, *P* 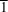
Temperature (K)	193
*a*, *b*, *c* (Å)	9.8368 (14), 10.2783 (15), 13.2857 (18)
α, β, γ (°)	97.689 (9), 108.305 (8), 108.103 (8)
*V* (Å^3^)	1171.6 (3)
*Z*	2
Radiation type	Cu *K*α
μ (mm^−1^)	5.20
Crystal size (mm)	0.40 × 0.20 × 0.08

Data collection	
Diffractometer	Enraf–Nonius CAD-4
Absorption correction	Numerical (*CORINC*; Dräger & Gattow, 1971 ▸)
*T*_min_, *T*_max_	0.24, 0.68
No. of measured, independent and observed [*I* > 2σ(*I*)] reflections	4706, 4431, 4039
*R*_int_	0.029
(sin θ/λ)_max_ (Å^−1^)	0.609

Refinement	
*R*[*F* ^2^ > 2σ(*F* ^2^)], *wR*(*F* ^2^), *S*	0.038, 0.107, 1.02
No. of reflections	4431
No. of parameters	299
H-atom treatment	H-atom parameters constrained
Δρ_max_, Δρ_min_ (e Å^−3^)	0.49, −0.42

Computer programs: *CAD-4 Software* (Enraf–Nonius, 1989 ▸), *CORINC* (Dräger & Gattow, 1971 ▸), *SHELXT* (Sheldrick, 2015*a* ▸), *SHELXL2018* (Sheldrick, 2015*b* ▸) and *PLATON* (Spek, 2020 ▸).

## full crystallographic data

### Crystal data

**Table:**

C_24_H_17_NO_2_S_3_·CH_2_Cl_2_	*Z* = 2
*M**_r_* = 532.49	*F*(000) = 548
Triclinic, *P*1	*D*_x_ = 1.509 Mg m^−^^3^
*a* = 9.8368 (14) Å	Cu *K*α radiation, λ = 1.54178 Å
*b* = 10.2783 (15) Å	Cell parameters from 25 reflections
*c* = 13.2857 (18) Å	θ = 65–70°
α = 97.689 (9)°	µ = 5.20 mm^−^^1^
β = 108.305 (8)°	*T* = 193 K
γ = 108.103 (8)°	Plate, red
*V* = 1171.6 (3) Å^3^	0.40 × 0.20 × 0.08 mm

### Data collection

**Table:**

Enraf–Nonius CAD-4 diffractometer	4039 reflections with *I* > 2σ(*I*)
Radiation source: rotating anode	*R*_int_ = 0.029
Graphite monochromator	θ_max_ = 70.0°, θ_min_ = 3.6°
ω/2θ scans	*h* = −11→0
Absorption correction: numerical (CORINC; Dräger & Gattow, 1971)	*k* = −11→12
*T*_min_ = 0.24, *T*_max_ = 0.68	*l* = −15→16
4706 measured reflections	3 standard reflections every 60 min
4431 independent reflections	intensity decay: 2%

### Refinement

**Table:**

Refinement on *F*^2^	Primary atom site location: structure-invariant direct methods
Least-squares matrix: full	Hydrogen site location: inferred from neighbouring sites
*R*[*F*^2^ > 2σ(*F*^2^)] = 0.038	H-atom parameters constrained
*wR*(*F*^2^) = 0.107	*w* = 1/[σ^2^(*F*_o_^2^) + (0.0626*P*)^2^ + 0.8284*P*] where *P* = (*F*_o_^2^ + 2*F*_c_^2^)/3
*S* = 1.02	(Δ/σ)_max_ < 0.001
4431 reflections	Δρ_max_ = 0.49 e Å^−^^3^
299 parameters	Δρ_min_ = −0.42 e Å^−^^3^
0 restraints	

### Special details

**Table:**

Geometry. All esds (except the esd in the dihedral angle between two l.s. planes) are estimated using the full covariance matrix. The cell esds are taken into account individually in the estimation of esds in distances, angles and torsion angles; correlations between esds in cell parameters are only used when they are defined by crystal symmetry. An approximate (isotropic) treatment of cell esds is used for estimating esds involving l.s. planes.

### Fractional atomic coordinates and isotropic or equivalent isotropic displacement parameters (Å^2^)

**Table:**

	*x*	*y*	*z*	*U*_iso_*/*U*_eq_	
N1	0.30399 (19)	0.17913 (18)	0.10488 (13)	0.0247 (4)	
C2	0.3290 (2)	0.2383 (2)	0.01978 (15)	0.0225 (4)	
C3	0.4616 (2)	0.2740 (2)	−0.00101 (16)	0.0257 (4)	
H3	0.546346	0.256542	0.044845	0.031*	
C4	0.4787 (2)	0.3357 (2)	−0.08731 (16)	0.0260 (4)	
S5	0.32556 (6)	0.36665 (6)	−0.17392 (4)	0.02863 (14)	
C6	0.1753 (2)	0.3113 (2)	−0.12982 (16)	0.0233 (4)	
C7	0.1890 (2)	0.2571 (2)	−0.04071 (15)	0.0217 (4)	
C8	0.0808 (2)	0.2101 (2)	0.01257 (16)	0.0232 (4)	
C9	−0.0709 (2)	0.2027 (2)	−0.01054 (17)	0.0274 (4)	
H9	−0.121736	0.232427	−0.071261	0.033*	
C10	−0.1450 (3)	0.1512 (2)	0.05678 (19)	0.0325 (5)	
H10	−0.248258	0.144580	0.041628	0.039*	
C11	−0.0704 (3)	0.1089 (2)	0.1464 (2)	0.0343 (5)	
H11	−0.123337	0.075406	0.192183	0.041*	
C12	0.0799 (3)	0.1145 (2)	0.17078 (18)	0.0306 (5)	
H12	0.130386	0.085282	0.231982	0.037*	
C13	0.1528 (2)	0.1643 (2)	0.10218 (16)	0.0242 (4)	
S14	0.43707 (6)	0.13882 (5)	0.19537 (4)	0.02610 (14)	
O15	0.51358 (18)	0.08036 (17)	0.13778 (13)	0.0340 (4)	
O16	0.35700 (18)	0.05745 (16)	0.25269 (13)	0.0348 (4)	
C17	0.5680 (2)	0.3040 (2)	0.28468 (16)	0.0255 (4)	
C18	0.7232 (2)	0.3448 (2)	0.30206 (17)	0.0292 (4)	
H18	0.758342	0.286801	0.262936	0.035*	
C19	0.8268 (2)	0.4717 (2)	0.37757 (18)	0.0300 (5)	
H19	0.933551	0.500361	0.389998	0.036*	
C20	0.7767 (2)	0.5579 (2)	0.43548 (17)	0.0283 (4)	
C21	0.6195 (3)	0.5153 (2)	0.41441 (17)	0.0296 (4)	
H21	0.583515	0.574240	0.451815	0.036*	
C22	0.5146 (2)	0.3889 (2)	0.34013 (17)	0.0293 (4)	
H22	0.407732	0.360408	0.327132	0.035*	
C23	0.8899 (3)	0.6957 (3)	0.5167 (2)	0.0407 (6)	
H23A	0.864225	0.774195	0.492812	0.061*	
H23B	0.994618	0.707837	0.521106	0.061*	
H23C	0.884507	0.694728	0.589017	0.061*	
S24	0.63754 (6)	0.38421 (7)	−0.11578 (5)	0.03617 (16)	
C25	0.0371 (2)	0.3359 (2)	−0.19783 (16)	0.0241 (4)	
C26	0.0170 (2)	0.4602 (2)	−0.16453 (17)	0.0296 (4)	
H26	0.086969	0.525949	−0.096614	0.035*	
C27	−0.1056 (3)	0.4880 (2)	−0.23081 (19)	0.0314 (5)	
H27	−0.119603	0.572988	−0.208273	0.038*	
C28	−0.2076 (2)	0.3919 (2)	−0.32976 (18)	0.0314 (5)	
H28	−0.291812	0.410854	−0.374891	0.038*	
C29	−0.1870 (3)	0.2687 (2)	−0.36283 (19)	0.0347 (5)	
H29	−0.257127	0.203008	−0.430764	0.042*	
C30	−0.0641 (2)	0.2401 (2)	−0.29718 (18)	0.0307 (5)	
H30	−0.049704	0.155542	−0.320298	0.037*	
C1L	0.4146 (3)	0.1202 (3)	0.6269 (2)	0.0428 (6)	
H1L1	0.497653	0.085655	0.659362	0.051*	
H1L2	0.444766	0.217600	0.670815	0.051*	
Cl1	0.39459 (7)	0.12334 (6)	0.49086 (4)	0.03707 (15)	
Cl2	0.24155 (10)	0.01018 (8)	0.63246 (7)	0.0643 (2)	

### Atomic displacement parameters (Å^2^)

**Table:**

	*U*^11^	*U*^22^	*U*^33^	*U*^12^	*U*^13^	*U*^23^
N1	0.0217 (8)	0.0301 (9)	0.0224 (8)	0.0102 (7)	0.0071 (7)	0.0091 (7)
C2	0.0227 (9)	0.0237 (9)	0.0193 (9)	0.0093 (8)	0.0057 (7)	0.0043 (7)
C3	0.0196 (9)	0.0334 (11)	0.0231 (10)	0.0117 (8)	0.0053 (8)	0.0071 (8)
C4	0.0223 (10)	0.0309 (10)	0.0223 (9)	0.0099 (8)	0.0068 (8)	0.0031 (8)
S5	0.0234 (3)	0.0407 (3)	0.0251 (3)	0.0134 (2)	0.0100 (2)	0.0135 (2)
C6	0.0208 (9)	0.0260 (9)	0.0217 (9)	0.0094 (8)	0.0066 (8)	0.0039 (7)
C7	0.0186 (9)	0.0221 (9)	0.0214 (9)	0.0068 (7)	0.0061 (7)	0.0025 (7)
C8	0.0215 (9)	0.0231 (9)	0.0227 (9)	0.0069 (8)	0.0083 (8)	0.0025 (7)
C9	0.0235 (10)	0.0272 (10)	0.0293 (10)	0.0101 (8)	0.0087 (8)	0.0016 (8)
C10	0.0233 (10)	0.0323 (11)	0.0392 (12)	0.0074 (9)	0.0148 (9)	0.0009 (9)
C11	0.0353 (12)	0.0315 (11)	0.0389 (12)	0.0082 (9)	0.0228 (10)	0.0065 (9)
C12	0.0332 (11)	0.0310 (11)	0.0290 (10)	0.0106 (9)	0.0146 (9)	0.0088 (8)
C13	0.0230 (10)	0.0222 (9)	0.0251 (9)	0.0070 (7)	0.0088 (8)	0.0026 (7)
S14	0.0270 (3)	0.0274 (3)	0.0231 (2)	0.0119 (2)	0.0059 (2)	0.00851 (19)
O15	0.0368 (9)	0.0359 (8)	0.0311 (8)	0.0212 (7)	0.0083 (7)	0.0065 (6)
O16	0.0364 (9)	0.0340 (8)	0.0311 (8)	0.0104 (7)	0.0087 (7)	0.0158 (6)
C17	0.0249 (10)	0.0295 (10)	0.0205 (9)	0.0101 (8)	0.0059 (8)	0.0086 (8)
C18	0.0282 (11)	0.0358 (11)	0.0283 (10)	0.0171 (9)	0.0108 (9)	0.0105 (9)
C19	0.0209 (10)	0.0388 (12)	0.0316 (11)	0.0132 (9)	0.0079 (8)	0.0131 (9)
C20	0.0268 (10)	0.0320 (11)	0.0246 (10)	0.0107 (9)	0.0063 (8)	0.0114 (8)
C21	0.0299 (11)	0.0347 (11)	0.0272 (10)	0.0136 (9)	0.0130 (9)	0.0080 (9)
C22	0.0228 (10)	0.0359 (11)	0.0294 (11)	0.0109 (9)	0.0107 (8)	0.0074 (9)
C23	0.0306 (12)	0.0359 (12)	0.0424 (13)	0.0075 (10)	0.0049 (10)	0.0022 (10)
S24	0.0232 (3)	0.0523 (3)	0.0324 (3)	0.0119 (2)	0.0123 (2)	0.0099 (2)
C25	0.0207 (9)	0.0302 (10)	0.0236 (9)	0.0107 (8)	0.0085 (8)	0.0105 (8)
C26	0.0259 (10)	0.0337 (11)	0.0267 (10)	0.0128 (9)	0.0067 (8)	0.0040 (8)
C27	0.0295 (11)	0.0330 (11)	0.0360 (12)	0.0171 (9)	0.0121 (9)	0.0101 (9)
C28	0.0203 (10)	0.0405 (12)	0.0357 (11)	0.0133 (9)	0.0093 (9)	0.0157 (9)
C29	0.0235 (10)	0.0380 (12)	0.0294 (11)	0.0072 (9)	0.0000 (9)	0.0023 (9)
C30	0.0262 (11)	0.0293 (10)	0.0321 (11)	0.0108 (9)	0.0059 (9)	0.0051 (9)
C1L	0.0435 (14)	0.0464 (14)	0.0294 (12)	0.0120 (11)	0.0072 (10)	0.0091 (10)
Cl1	0.0385 (3)	0.0410 (3)	0.0320 (3)	0.0176 (2)	0.0107 (2)	0.0099 (2)
Cl2	0.0688 (5)	0.0593 (4)	0.0611 (5)	0.0072 (4)	0.0338 (4)	0.0209 (4)

### Geometric parameters (Å, º)

**Table:**

N1—C2	1.405 (3)	C18—C19	1.389 (3)
N1—C13	1.436 (3)	C18—H18	0.9500
N1—S14	1.6771 (17)	C19—C20	1.395 (3)
C2—C3	1.367 (3)	C19—H19	0.9500
C2—C7	1.446 (3)	C20—C21	1.391 (3)
C3—C4	1.412 (3)	C20—C23	1.506 (3)
C3—H3	0.9500	C21—C22	1.382 (3)
C4—S24	1.665 (2)	C21—H21	0.9500
C4—S5	1.732 (2)	C22—H22	0.9500
S5—C6	1.721 (2)	C23—H23A	0.9800
C6—C7	1.361 (3)	C23—H23B	0.9800
C6—C25	1.494 (3)	C23—H23C	0.9800
C7—C8	1.453 (3)	C25—C30	1.386 (3)
C8—C9	1.401 (3)	C25—C26	1.388 (3)
C8—C13	1.402 (3)	C26—C27	1.388 (3)
C9—C10	1.381 (3)	C26—H26	0.9500
C9—H9	0.9500	C27—C28	1.385 (3)
C10—C11	1.391 (3)	C27—H27	0.9500
C10—H10	0.9500	C28—C29	1.380 (3)
C11—C12	1.391 (3)	C28—H28	0.9500
C11—H11	0.9500	C29—C30	1.391 (3)
C12—C13	1.383 (3)	C29—H29	0.9500
C12—H12	0.9500	C30—H30	0.9500
S14—O15	1.4257 (16)	C1L—Cl2	1.757 (3)
S14—O16	1.4274 (16)	C1L—Cl1	1.761 (2)
S14—C17	1.758 (2)	C1L—H1L1	0.9900
C17—C18	1.384 (3)	C1L—H1L2	0.9900
C17—C22	1.391 (3)		
			
C2—N1—C13	109.41 (16)	C17—C18—C19	119.0 (2)
C2—N1—S14	123.30 (14)	C17—C18—H18	120.5
C13—N1—S14	127.27 (14)	C19—C18—H18	120.5
C3—C2—N1	126.56 (18)	C18—C19—C20	121.1 (2)
C3—C2—C7	126.24 (18)	C18—C19—H19	119.5
N1—C2—C7	107.19 (17)	C20—C19—H19	119.5
C2—C3—C4	123.21 (18)	C21—C20—C19	118.5 (2)
C2—C3—H3	118.4	C21—C20—C23	120.8 (2)
C4—C3—H3	118.4	C19—C20—C23	120.6 (2)
C3—C4—S24	125.67 (16)	C22—C21—C20	121.3 (2)
C3—C4—S5	119.70 (15)	C22—C21—H21	119.4
S24—C4—S5	114.63 (12)	C20—C21—H21	119.4
C6—S5—C4	107.18 (10)	C21—C22—C17	119.0 (2)
C7—C6—C25	126.27 (18)	C21—C22—H22	120.5
C7—C6—S5	121.92 (15)	C17—C22—H22	120.5
C25—C6—S5	111.76 (14)	C20—C23—H23A	109.5
C6—C7—C2	121.71 (18)	C20—C23—H23B	109.5
C6—C7—C8	130.91 (18)	H23A—C23—H23B	109.5
C2—C7—C8	107.38 (17)	C20—C23—H23C	109.5
C9—C8—C13	119.53 (19)	H23A—C23—H23C	109.5
C9—C8—C7	132.47 (19)	H23B—C23—H23C	109.5
C13—C8—C7	108.00 (17)	C30—C25—C26	120.25 (19)
C10—C9—C8	118.6 (2)	C30—C25—C6	120.14 (18)
C10—C9—H9	120.7	C26—C25—C6	119.45 (18)
C8—C9—H9	120.7	C27—C26—C25	119.8 (2)
C9—C10—C11	120.9 (2)	C27—C26—H26	120.1
C9—C10—H10	119.6	C25—C26—H26	120.1
C11—C10—H10	119.6	C28—C27—C26	120.1 (2)
C10—C11—C12	121.6 (2)	C28—C27—H27	120.0
C10—C11—H11	119.2	C26—C27—H27	120.0
C12—C11—H11	119.2	C29—C28—C27	120.0 (2)
C13—C12—C11	117.3 (2)	C29—C28—H28	120.0
C13—C12—H12	121.4	C27—C28—H28	120.0
C11—C12—H12	121.4	C28—C29—C30	120.3 (2)
C12—C13—C8	122.10 (19)	C28—C29—H29	119.8
C12—C13—N1	129.92 (19)	C30—C29—H29	119.8
C8—C13—N1	107.98 (17)	C25—C30—C29	119.5 (2)
O15—S14—O16	120.02 (10)	C25—C30—H30	120.2
O15—S14—N1	108.03 (9)	C29—C30—H30	120.2
O16—S14—N1	105.28 (9)	Cl2—C1L—Cl1	111.26 (14)
O15—S14—C17	109.01 (10)	Cl2—C1L—H1L1	109.4
O16—S14—C17	109.45 (10)	Cl1—C1L—H1L1	109.4
N1—S14—C17	103.79 (9)	Cl2—C1L—H1L2	109.4
C18—C17—C22	121.13 (19)	Cl1—C1L—H1L2	109.4
C18—C17—S14	119.48 (16)	H1L1—C1L—H1L2	108.0
C22—C17—S14	119.34 (16)		
			
C13—N1—C2—C3	176.94 (19)	C2—N1—C13—C8	2.0 (2)
S14—N1—C2—C3	−1.4 (3)	S14—N1—C13—C8	−179.75 (14)
C13—N1—C2—C7	−2.0 (2)	C2—N1—S14—O15	−39.63 (18)
S14—N1—C2—C7	179.68 (13)	C13—N1—S14—O15	142.36 (17)
N1—C2—C3—C4	−178.83 (18)	C2—N1—S14—O16	−169.00 (16)
C7—C2—C3—C4	−0.1 (3)	C13—N1—S14—O16	12.99 (19)
C2—C3—C4—S24	178.86 (16)	C2—N1—S14—C17	76.01 (17)
C2—C3—C4—S5	−1.0 (3)	C13—N1—S14—C17	−102.00 (18)
C3—C4—S5—C6	0.5 (2)	O15—S14—C17—C18	−10.2 (2)
S24—C4—S5—C6	−179.37 (11)	O16—S14—C17—C18	122.83 (17)
C4—S5—C6—C7	1.1 (2)	N1—S14—C17—C18	−125.19 (17)
C4—S5—C6—C25	178.86 (14)	O15—S14—C17—C22	172.41 (16)
C25—C6—C7—C2	−179.66 (18)	O16—S14—C17—C22	−54.52 (19)
S5—C6—C7—C2	−2.2 (3)	N1—S14—C17—C22	57.47 (18)
C25—C6—C7—C8	−0.2 (3)	C22—C17—C18—C19	1.0 (3)
S5—C6—C7—C8	177.20 (16)	S14—C17—C18—C19	−176.29 (16)
C3—C2—C7—C6	1.9 (3)	C17—C18—C19—C20	−0.1 (3)
N1—C2—C7—C6	−179.21 (17)	C18—C19—C20—C21	−1.3 (3)
C3—C2—C7—C8	−177.69 (19)	C18—C19—C20—C23	−179.6 (2)
N1—C2—C7—C8	1.2 (2)	C19—C20—C21—C22	1.7 (3)
C6—C7—C8—C9	1.2 (4)	C23—C20—C21—C22	−179.9 (2)
C2—C7—C8—C9	−179.3 (2)	C20—C21—C22—C17	−0.8 (3)
C6—C7—C8—C13	−179.5 (2)	C18—C17—C22—C21	−0.6 (3)
C2—C7—C8—C13	0.0 (2)	S14—C17—C22—C21	176.71 (16)
C13—C8—C9—C10	0.5 (3)	C7—C6—C25—C30	−100.3 (3)
C7—C8—C9—C10	179.7 (2)	S5—C6—C25—C30	82.0 (2)
C8—C9—C10—C11	0.7 (3)	C7—C6—C25—C26	84.2 (3)
C9—C10—C11—C12	−1.1 (3)	S5—C6—C25—C26	−93.4 (2)
C10—C11—C12—C13	0.2 (3)	C30—C25—C26—C27	0.4 (3)
C11—C12—C13—C8	1.1 (3)	C6—C25—C26—C27	175.86 (19)
C11—C12—C13—N1	−178.4 (2)	C25—C26—C27—C28	0.1 (3)
C9—C8—C13—C12	−1.4 (3)	C26—C27—C28—C29	−0.3 (3)
C7—C8—C13—C12	179.21 (18)	C27—C28—C29—C30	0.0 (3)
C9—C8—C13—N1	178.17 (17)	C26—C25—C30—C29	−0.7 (3)
C7—C8—C13—N1	−1.2 (2)	C6—C25—C30—C29	−176.1 (2)
C2—N1—C13—C12	−178.4 (2)	C28—C29—C30—C25	0.4 (3)
S14—N1—C13—C12	−0.2 (3)		
